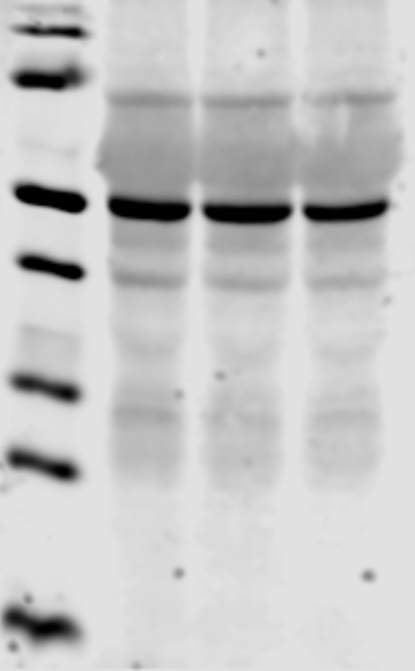# Curcumin simultaneously improves mitochondrial dynamics and myocardial cell bioenergy after sepsis via the SIRT1-DRP1/PGC-1α pathway

**DOI:** 10.1016/j.heliyon.2024.e28501

**Published:** 2024-03-28

**Authors:** Dongyao Hou, Haitang Liao, Shuai Hao, Ruixue Liu, He Huang, Chenyang Duan

**Affiliations:** a Department of Anesthesiology, The Second Affiliated Hospital of Chongqing Medical University, Chongqing, 400010, China; b Department of Anesthesiology, Taihe Hospital, Hubei University of Medicine, Shiyan, 442000, China; c Department of Intensive Care Unit, Chongqing Hospital of Traditional Chinese Medicine, Chongqing, 400011, China; d Research Institute of General Surgery, Jinling Hospital, Medical School of Nanjing University, Nanjing, 210002, China

**Keywords:** Cardiac dysfunction, Mitochondrial biogenesis, Oxidative stress, Inflammation

## Abstract

Septic cardiomyopathy (SCM) is associated with an imbalance in mitochondrial quality and high mortality rates, with no effective treatment developed to date. Curcumin provides antioxidant, anti-inflammatory, cardiovascular, and mitochondrial protection. However, curcumin has not been confirmed to improve cardiac dysfunction in sepsis. We hypothesized that curcumin can reduce abnormal inflammatory responses by improving mitochondrial function as a novel mechanism to improve SCM. To explore this hypothesis, we used an *in vivo* male C57BL/6 mouse sepsis model and an *in vitro* model of lipopolysaccharide-stimulated HL-1 cells. The effects of curcumin on sepsis-induced cardiac dysfunction, inflammatory responses, and mitochondrial quality of cardiac cells were observed using quantitative polymerase chain reaction, western blotting, echocardiography, and transmission electron microscopy. Curcumin activated sirtuin 1 (SIRT1); increased expression of the mitochondrial biogenesis-related genes *Pgc1α*, *Tfam*, and *Nrf2;* reduced dynamin-related protein 1 translocation from the cytoplasm to mitochondria; and restored the mitochondrial morphology and function in cardiac cells. Accordingly, curcumin protected heart function after septic shock and alleviated the effects of SCM. SIRT1 knockdown reversed the protective effects of curcumin on mitochondria. Therefore, curcumin promotes mitochondrial biogenesis and inhibits mitochondrial fragmentation by activating SIRT1, thereby improving the mitochondrial quality and reducing oxidative stress in cardiomyocytes and sepsis-induced cardiac dysfunction. These findings provide new evidence supporting the use of curcumin to treat SCM.

## 1 Introduction

Sepsis is a life-threatening organ dysfunction caused by dysregulation of the body’s response to infection. Millions of patients are newly diagnosed with sepsis worldwide annually, and this condition leads to the death of 1/6 to 1/3 of these patients. However, early recognition and appropriate treatment within the first few hours after the onset of sepsis can improve the prognosis [1]. Septic cardiomyopathy (SCM) is one of the most severe and challenging complications to treat, with approximately 50% of patients with sepsis developing SCM, and the mortality rates reach up to 70%–90% [2]. The pathological process of SCM may involve various mechanisms, including tissue hypoxia, decreased contractile function, and impaired energy metabolism [3]. However, no effective drugs have been developed to treat SCM to date.

Mitochondrial quality imbalance and dysfunction are pathophysiological mechanisms of SCM [14]. Mitochondria are the primary sites for aerobic respiration and also play a critical role in providing metabolites [4,5] and regulating cell signaling [[6], [7], [8]], calcium uptake [9,10], and cell death [[11], [12], [13]]. Under normal physiological conditions, mitochondrial quality control is essential for maintaining mitochondrial homeostasis and function, including mitochondrial biogenesis, fusion/fission, and mitophagy. Mitochondrial biogenesis is a regulated process involving nuclear and mitochondrial genome coordination [15]. Furthermore, mitochondrial membrane potential (ΔΨm) is a crucial component directly influencing mitochondrial function, and reactive oxygen species (ROS) production is closely associated with mitochondrial function. Among these regulatory mechanisms, peroxisome proliferator-activated receptor gamma coactivator-1α (PGC-1α) is a major regulator that activates the downstream mitochondrial transcription factor TFAM to encode mitochondria-specific proteins [16]. Damage to mitochondrial biogenesis may lead to dysfunction and impact cardiac dysfunction pathogenesis in sepsis [17]. In addition, mitochondrial fission and fusion play crucial roles in maintaining mitochondrial quality under conditions of altered energy demand and environmental stress [18]. In SCM, excessive dynamin-related protein 1 (DRP1) accumulation in the mitochondria promotes mitochondrial fragmentation. In contrast, inhibition of myocardial mitochondrial fission can prevent a decline in cardiac function and reduce mortality [19].

The abnormal inflammatory response caused by oxidative stress is the core event of the occurrence and development of cardiac dysfunction in sepsis. Curcumin is a polyphenolic antioxidant extracted from *Curcuma longa* rhizomes. Owing to its hydrophobic nature, curcumin can diffuse through cell membranes to act on the endoplasmic reticulum, mitochondria, and nucleus [20] and has been found to have anti-inflammatory, anti-apoptotic, anti-fibrotic, anti-tumor, and cardiovascular protection in addition to its antioxidant effects [3,21]. Although curcumin has protective effects against myocardial injury caused by sepsis, reducing myocardial inflammation and alleviating structural damage to myocardial cells in sepsis [22], its potential therapeutic value in exerting mitochondrial protective effects during sepsis and the underlying mechanism remain unclear.

Therefore, the aim of this study was to investigate the protective effects of curcumin against lipopolysaccharide (LPS)-induced cardiac dysfunction in mouse and cellular sepsis models, particularly focusing on whether curcumin can regulate myocardial function by improving mitochondrial quality. These findings serve as a vital reference for the potential application of curcumin as a drug for treating SCM.

## 2 Materials and methods

### 2.1 Animals and drug treatment

Male C57BL/6J wild-type mice (6 weeks old, body weight 20.3 ± 4.4 g) were obtained from the Experimental Animal Center of Chongqing Medical University. Mice were acclimated for at least 1 week under a controlled temperature of 22 °C with 40–70% humidity and a 12-h light/dark cycle with *ad libitum* access to standard pelleted rodent chow and filtered water. Food was restricted for 12 h before the experiment, but water was freely available to the mice. The mice were randomly divided into control (CON) and LPS groups (LPS derived from *Escherichia coli* 055:B5). The CON group received tap water and standard laboratory chow. The LPS group was further divided into two subgroups: one subgroup was intraperitoneally injected with LPS (10 mg/kg), and the other subgroup (Cur group) was injected with LPS + curcumin (80 mg/kg) (peros) [23]. Curcumin was administered orally once daily at a volume of 4 mL/kg body weight between 14:00 and 15:00 for 4 weeks. Mice were decapitated 24 h after LPS stimulation to obtain plasma and cardiac tissues. The survival rate was monitored every 2 h for 48 h after LPS stimulation.

MT-CYB (#54618) was purchased from Cell Signaling Technology (Danvers, MA, USA). Acetyl-lysine (PTM-101) was obtained from Jingjie Biotech (Hangzhou, China). TFAM (ab252432) and mitochondrial cytochrome C oxidase II (MT-CO2) (ab198286) were purchased from Abcam (Cambridge, USA). β-Tubulin antibody (sc-5274) was purchased from Santa Cruz Biotechnology (Santa Cruz, CA, USA). Pierce™ Co-Immunoprecipitation Kit (26149) was purchased from Invitrogen (Carlsbad, CA, USA). RIPA buffer (P0013B), protease inhibitor cocktail (P1045-1), phosphatase inhibitor cocktail (P1045-2), and ECGS were purchased from Millipore (Darmstadt, Germany). Recombinant trypsin (P4201) and type II collagenase (ST2303) were purchased from BioSharp (Shanghai, China). A Pro-Light HRP Chemiluminescence Kit (PA112) was purchased from Tiangen Biotech (Beijing, China). Unless otherwise specified, all other chemicals were purchased from Sigma-Aldrich.

### 2.2 Echocardiographic examination

Post-anesthetized mice were subjected to transthoracic echocardiographic examination using the VIDIVE 9 High-Frequency Color Doppler Ultrasound System (GE Healthcare, Boston, MA, USA) to evaluate their cardiac function. M-mode echocardiography was performed in the long-axis view of the left ventricle to assess systolic function. Left ventricular functional parameters, including fractional shortening and ejection fraction, were calculated.

### 2.3 Histological examination

The heart was stained with hematoxylin and eosin (H&E) according to previously reported methods [23]. The obtained heart specimens were fixed in 4% paraformaldehyde, embedded in paraffin, and sliced to a thickness of 5 μm. Left ventricular specimens were stained with H&E and examined under an optical microscope (Leica DMi8).

### 2.4 Biochemical analysis

The eyes of the mice were removed, and 500 μL blood samples were collected. The samples were allowed to stand at 20 ± 2 °C for 1 h and then centrifuged at 3000 rpm for 5 min to obtain 100 μL serum. ELISA kits (E-MSEL-M0001, KitE-MSEL-M0002, and E-MSEL-M0003; Elabscience, Wuhan, China) were used to measure the levels of IL-6, tumor necrosis factor-alpha (TNF-α), and IL-1β in the mouse serum.

### 2.5 Transmission electron microscopy

Heart samples were prepared as described previously [24]. The samples were observed using a transmission electron microscope (H-7500; Hitachi, Japan), and mitochondrial images were analyzed by a technician blinded to the treatment using ImageJ software (https://fiji.sc/).

### 2.6 Cell culture and treatment

HL-1 cells (a mouse cardiomyocyte cell line; Cell Bank of the Chinese Academy of Sciences, Shanghai, China) were cultured in Dulbecco’s modified Eagle medium (DMEM) supplemented with 10% fetal bovine serum, maintained at 37 °C, and cultured in a humidified environment of 5% CO_2_/95% air. When the cardiomyocytes reached over 70% confluence in the culture medium, they were randomly treated with DMEM (CON group), 1 μg/mL LPS (LPS group), 1 μg/mL LPS + 20 μM curcumin (Cur group), DMEM + 100 μM chloroquine phosphate (CQ) (CON + CQ group), 1 μg/mL LPS + 100 μM CQ (LPS + CQ group), or 1 μg/mL LPS + 20 μM curcumin +100 μM CQ (Cur + CQ group) for 24 h. After treatment, the cells were collected for relevant experiments.

### 2.7 Measurement of inflammatory genes and mitochondrial DNA

Inflammatory cytokines in the heart tissues [25] and mitochondrial DNA (mtDNA) content [26] were determined using reverse transcription-quantitative polymerase chain reaction (RT-qPCR) according to previously described methods. Total mRNA was extracted from mouse myocardial tissues or HL-1 cardiomyocytes using RNAiso Plus (Code No. 9108/9109; Takara, Japan), and cDNA was synthesized using the ABScript II cDNA First-Strand Synthesis Kit (RK20400, Abclonal, Wuhan, China). For mtDNA quantification, total DNA was extracted with the QIAamp DNA mini kit (QIAGENE, Germantown, MD) according to the manufacturer’s instructions and subjected to RT-qPCR to assess the relative levels of mitochondrial DNA D-Loop structure and mtDNA-encoded MT-CO2 versus genomic DNA-encoded beta-actin. All procedures were performed according to the manufacturer’s instructions. The primer sequences are shown in Table 1.

**Table 1:** Sequences of all primer pairs used in the study.

Gene Name	Primer sequence
IL-6-F	TGAGAAAAGAGTTGTGCAATGG
IL-6-R	GGTACTCCAGAAGACCAGAGG
TNF-α-F	GGTGCCTATGTCTCAGCCTC
TNF-α-R	GCTCCTCCACTTGGTGGTTT
IL-β-F	TGCCACCTTTTGACAGTGATG
IL-β-R	TGATGTGCTGCTGCGAGATT
D-Loop-F	GATTTGGGTACCACCCAAGTATTG
D-Loop-R	GTACAATATTCATGGTGGCTGGCA
MT-CO2-F	CCTGCGACTCCTTGACGTTG
MT-CO2-R	AGCGGTGAAAGTGGTTTGGTT
MT-CYB-F	ATCACTCGAGACGTAAATTATGGCT
MT-CYB-R	TGAACTAGGTCTGTCCCAATGTATG
GAPDH-F	TGAAGGTCGGTGTGAACGG
GAPDH-R	GTGAGTGGAGTCATACTGGAA

### 2.8 Adenovirus and short hairpin RNA (shRNA) transfection

The plasmids for SIRT1 shRNA (sh-SIRT1) and scrambled shRNA were obtained from GeneChem, and their sequences are shown in Table 2. HL-1 cells were transfected with shRNA plasmids using Lipofectamine™ 3000 (Invitrogen, Carlsbad, CA, USA) lipid reagent, following the manufacturer’s instructions. Twenty-four hours after the shRNA transfection, the cells were treated as described in Section 2.6. The shRNA sequences are shown in Table 2.

**Table 2:** Sequences of sh-SIRT1 and sh-NC mRNA.

Name	shRNA sequence
sh-SIRT1	ACCGGGATGCTGTGAAGTTACTGCTACTCGAGTAGCAGTAACTTCACAGCATCTTTTTTGAATTC
sh-NC	ACCGGCCTAAGGTTAAGTCGCCCTCGCTGAGCGAGGGCGACTTAACCTTAGGTTTTTGAATTC

### 2.9 Morphology, ROS, and ΔΨm assays

HL-1 cells were seeded into 20-mm-diameter confocal culture dishes and then treated as described in Section 2.6. Once the cell seeding density reached 50%, the cells were washed with 1 × phosphate-buffered saline (PBS). MitoTracker Deepred (100 nM; M22426; Invitrogen; Carlsbad, CA, USA), JC-1 staining working solution (10 μg/mL; C2006; Millipore, Darmstadt, Germany) [28], or DCFH-DA ROS fluorescent probe (10 μM; S0033S; Millipore, Darmstadt, Germany) was added to the cells [29], which were then incubated at 37 °C in a 5% CO_2_ incubator for 30 min. Cells were imaged using laser confocal microscopy (Leica TCS SP5, Germany). ROS fluorescence and JC-1 monomers were excited using a 488 nm laser and collected at 501–563 nm emission, whereas MitoTracker and JC-1 aggregate fluorescence were excited using a 633 nm laser and collected at 558–617 nm emission. Images were quantified using ImageJ software (https://fiji.sc/).

### 2.10 Western blot analysis

Proteins were extracted from fresh tissues and HL-1 cells, and total protein expression was measured as previously described [24]. To analyze mitochondrial and cytoplasmic protein expression, HL-1 cells were collected in centrifuge tubes, and cytoplasmic and mitochondrial fractions were isolated using a cytoplasmic and mitochondrial isolation kit (SC-003 and MP-007, respectively; Invent Biotechnologies, Beijing, China) according to the manufacturer’s instructions [27]. Proteins of isolated subcellular fractions (approximately 20 μg) were separated on a 10% sodium dodecyl sulfate-polyacrylamide gel and transferred onto a polyvinylidene fluoride membrane. The membranes were blocked in Tris buffer containing 5% skim milk, 1% BSA, and 0.1% Tween-20 for 1 h. The membrane was incubated overnight with primary antibodies against PGC-1α (#2178; dilution 1:1000; Cell Signaling Technology, Danvers, MA, USA), SIRT1 (ab189494; dilution 1:1000; Abcam, Cambridge, UK), DRP1 (ab56788; dilution 1:1000; Abcam, Cambridge, UK), COX IV (ab110272; dilution 1:2000; Abcam, Cambridge, UK), tubulin (dilution 1:5000), and GAPDH (ab59164; dilution 1:5000; Abcam, Cambridge, UK). After washing three times with 0.1% Tween-20 in Tris buffer for 15 min each, the membrane was incubated with secondary antibodies conjugated to horseradish peroxidase (A0216; dilution 1:2000; Millipore, Darmstadt, Germany) at room temperature for 45 min. The band intensity was analyzed using Quantity One V4.62 software (Bio-Rad, Life Science, USA). GAPDH, COX4, and β-tubulin were used as internal references for the total protein, mitochondrial, and cytoplasmic fractions, respectively. The relative expression level of the target protein was calculated as the ratio of the target protein gray value to the internal reference gray value using ImageJ software (https://fiji.sc/).

### 2.11 Co-immunoprecipitation

Co-immunoprecipitation (Co-IP) was performed using the Pierce Classic Magnetic IP/Co-IP Kit (Invitrogen, Carlsbad, CA, USA) according to the manufacturer’s instructions [30]. The sample was incubated with the PGC-1α antibody and subjected to Co-IP using the Protein A/G Magnetic Bead IP Kit. First, 10 μg of PGC-1α antibody was diluted in 200 μL of PBS with Tween-20, and 50 μL of Protein A/G Magnetic Beads were added to the mixture and incubated at room temperature for 10 min. The supernatant was removed to prepare PGC-1α-bound immunomagnetic beads. After collecting, lysing, and centrifuging (4 °C, 2500×*g*, 15 min) the samples, the supernatant was gently mixed with the PGC-1α-bound immunomagnetic beads to prepare the immunomagnetic bead–antibody–antigen complex. After three washes with PBS, the complex was resuspended in 100 μL of PBS to detect the endogenous interaction between Kac and PGC-1α by showing the intensity of the Kac band.

### 2.12 Statistical analysis

Statistical analyses were performed using SPSS software (version 18.0, SPSS Inc., Chicago, IL, USA). Data are presented as the mean ± standard deviation. Differences between multiple groups were compared using analysis of variance, followed by a post-hoc least-significant difference test for multiple comparisons. Survival analysis was performed using the Kaplan–Meier method. P < 0.05 was considered statistically significant.

## 3 Results

### 3.1 Curcumin improves survival of sepsis model mice by protecting cardiac function

We established a mouse model of sepsis by intraperitoneal LPS injection and evaluated the effect of curcumin on the survival of LPS-treated mice. The 24-h mortality rate of mice in the LPS group was 83% (10/12), while the 48-h rate was 100%; the mean survival time was 19.833 ± 7.5 h. In contrast, the 24-h mortality rate in the curcumin group was 25% (3/12), and the 48-h mortality rate was 75% (9/12); the mean survival duration was 34 ± 11.86 h. These findings indicate that curcumin treatment can significantly improve the survival rate of LPS-induced mice (Fig. 1a).

**Fig. 1:**
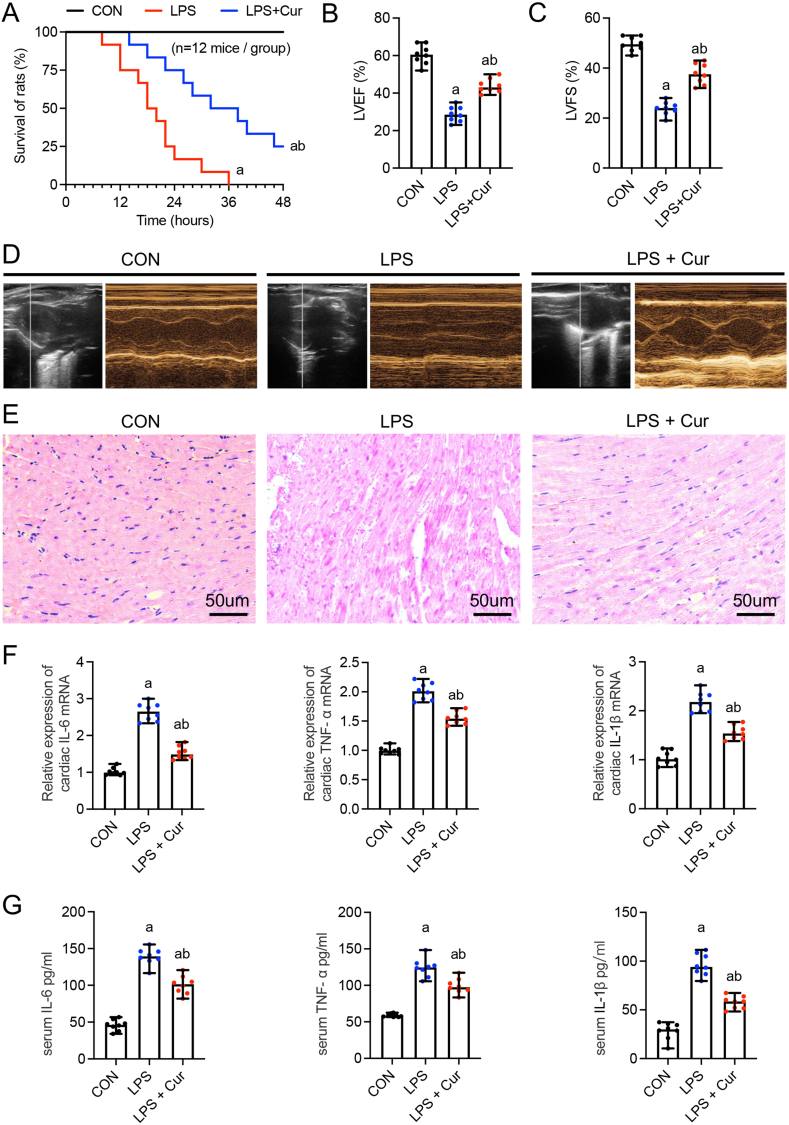
Protective effect of curcumin on septic mice induced by LPS. **(a)** Survival rate and time (n = 12). **(b)** Left ventricular ejection fraction (LVEF) (n = 8). **(c)** Left ventricular fractional shortening (LVFS) (n = 8). **(d)** Representative echocardiography images (scale bar, 50 μm). **(e)** Histochemical sections of the left ventricle stained with hematoxylin and eosin (scale bar, 50 μm). **(f)** Relative mRNA expression levels of inflammatory cytokines, including IL-6, TNF-α, and IL-1β, in the mouse hearts (n = 8). **(g)** Expression levels of peripheral blood inflammatory factors IL-6, TNF-α, and IL-1β in mice (n = 8). The data are presented as the mean ± standard deviation. a, Statistically significant difference (P < 0.05) compared to the control group; b, statistically significant difference (P < 0.05) compared to the LPS group.

To evaluate the effect of curcumin on cardiac function, we measured indicators related to myocardial contraction. Compared to those of the CON group, LPS treatment resulted in 31% and 25% reductions in the left ventricular ejection fraction (LVEF) and left ventricular fractional shortening (LVFS), respectively. However, these indicators improved after curcumin pretreatment, with a 15% and 14% increase in LVEF and LVFS, respectively (P < 0.05) (Fig. 1b and c). These findings suggest that curcumin significantly improves ventricular contractility after sepsis. Additionally, echocardiography revealed that cardiac contractile function was significantly improved in the curcumin group compared to that in the LPS group, and left ventricular structural abnormalities were reduced (Fig. 1d).

H&E staining showed that myocardial fibers were neatly arranged in the control group, and cardiomyocytes showed intact morphology. In contrast, the LPS group showed a disordered arrangement of myocardial fibers, partial blurring or disappearance of cross-striations, myocardial cell swelling, and widening of the interstitial spaces. However, these structural abnormalities were alleviated in the Cur group (Fig. 1e).

We further measured the relative mRNA expression levels of inflammatory cytokines in the mouse hearts. Compared with those in the LPS group, the relative expression levels of *Il6*, *Tnfα*, and *Il1β* mRNA in the Cur group were significantly decreased at 1.53 ± 0.16, 1.55 ± 0.10, and 1.55 ± 0.13, respectively (Fig. 1f). Inflammatory cytokine expression in the peripheral blood also showed a similar trend (Fig. 1g). These results suggest that curcumin can effectively inhibit TNF-α, IL-6, and IL-1β expression in the heart and serum of septic mice. Therefore, curcumin prolongs the survival of mice with sepsis, improves cardiac function, and reduces inflammatory responses.

### 3.2 Curcumin improves mitochondrial dynamics in septic cardiomyocytes by inhibiting the mitochondrial translocation of DRP1

To investigate whether the protective effect of curcumin on sepsis-induced cardiomyopathy was related to the mitochondria, we evaluated mitochondrial morphology in mice treated with LPS and curcumin. Transmission electron microscopy analysis revealed that the cardiomyocyte mitochondria in the CON group had regular arrangements and clear internal cristae structures. In contrast, the LPS group showed mitochondrial abnormalities, including an uneven distribution, swelling, cristae disintegration or loss, and severe lamellar vacuolization changes. However, after curcumin treatment, mitochondrial structural damage was significantly reduced compared to that in the LPS group, with clear cristae structures and a restored mitochondrial matrix (Fig. 2a). Additionally, we observed the mitochondrial morphology of cardiomyocytes in each group using confocal microscopy. The mitochondria in the HL-1 cells were primarily short and punctate after LPS treatment. After curcumin treatment, the mitochondrial morphology recovered from punctate to filamentous, and the mitochondrial reticular structure was partially restored (P < 0.05) (Fig. 2b). These findings suggest that curcumin alleviates the degree of mitochondrial fragmentation in cardiomyocytes after sepsis.

**Fig. 2:**
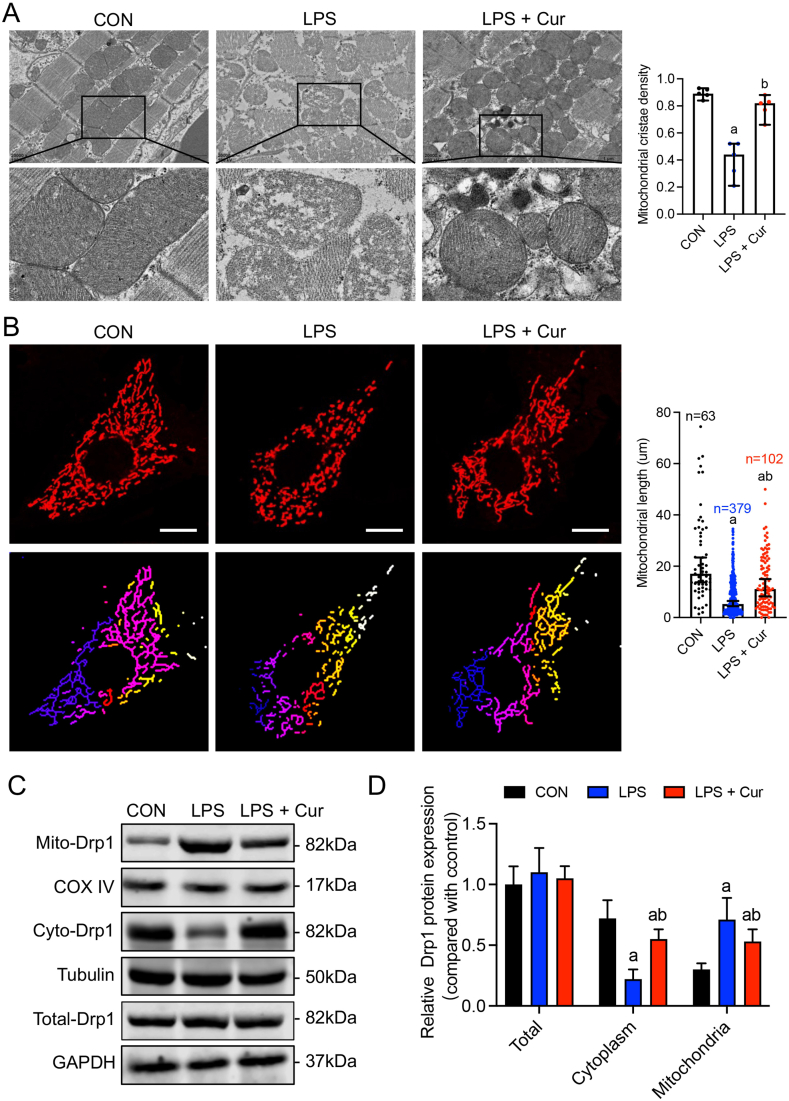
Effect of curcumin on the mitochondrial morphology of mouse cardiomyocytes induced by LPS. **(a)** Transmission electron microscopy images of mitochondrial morphology in cardiomyocytes (n = 5). **(b)** Confocal images (63 × magnification; scale bar, 25 μm) of mitochondrial morphology in HL-1 cells in each group analyzed for the mitochondrial skeleton using Image J software. **(c and d)** Effect of curcumin on mitochondrial, cytoplasmic, and total cellular DRP1 protein expression in LPS-induced HL-1 cells (n = 3). The data are presented as the mean ± standard deviation. a, Statistically significant difference (P < 0.05) compared to the control group; b, statistically significant difference (P < 0.05) compared to the LPS group.

To determine the reason for the changes in cardiomyocyte mitochondrial morphology after LPS stimulation, we measured indicators of mitochondrial dynamics. DRP1 is a classic mitochondrial dynamics protein that mainly affects mitochondrial fission during quality control [31]. Western blotting showed that the total DRP1 expression level in mouse cardiomyocytes did not significantly increase after LPS stimulation; however, the amount of DRP1 protein translocated from the cytoplasm to the mitochondria increased significantly. Compared to that in the CON group, DRP1 expression in cardiomyocyte mitochondria in the LPS group increased by 2-fold, and after curcumin treatment, mitochondrial DRP1 expression was significantly downregulated (Fig. 2c and d, Fig, S1). These data suggest that curcumin reduces the degree of mitochondrial fragmentation in cardiomyocytes after sepsis by inhibiting DRP1 translocation to the mitochondria, thereby improving cardiomyocyte mitochondrial morphology.

### 3.3 Curcumin improves mitochondrial biogenesis in cardiomyocytes after sepsis through activation of the PGC1 pathway

To investigate whether curcumin treatment improves cardiac function by promoting mitochondrial biogenesis, we measured the mtDNA content in mouse cardiac HL-1 cells. After LPS stimulation, the gene expression of the mtDNA-encoded proteins mtDNA D-loop structure, MT-CO2, and MT-CYB significantly decreased in HL-1 cells (Fig. 3a). However, after curcumin treatment, the expression levels of these genes increased by 15%, 27%, and 29%, respectively (P < 0.05). Western blotting confirmed that curcumin promoted the expression of mtDNA-encoded proteins, particularly MT-CO2 and MT-CYB (Fig. 3b and c, Fig, S2). Because LPS-induced mitophagy may affect mtDNA, we treated cells with CQ to inhibit autophagosome-lysosome fusion. CQ treatment partially reversed the LPS-mediated downregulation of mtDNA, MT-CO2, and MT-CYB (P > 0.05) (Fig. 3a–c, Fig, S2). This finding suggests that LPS stimulation inhibits mitochondrial biogenesis in cardiac cells independent of mitophagy.

**Fig. 3:**
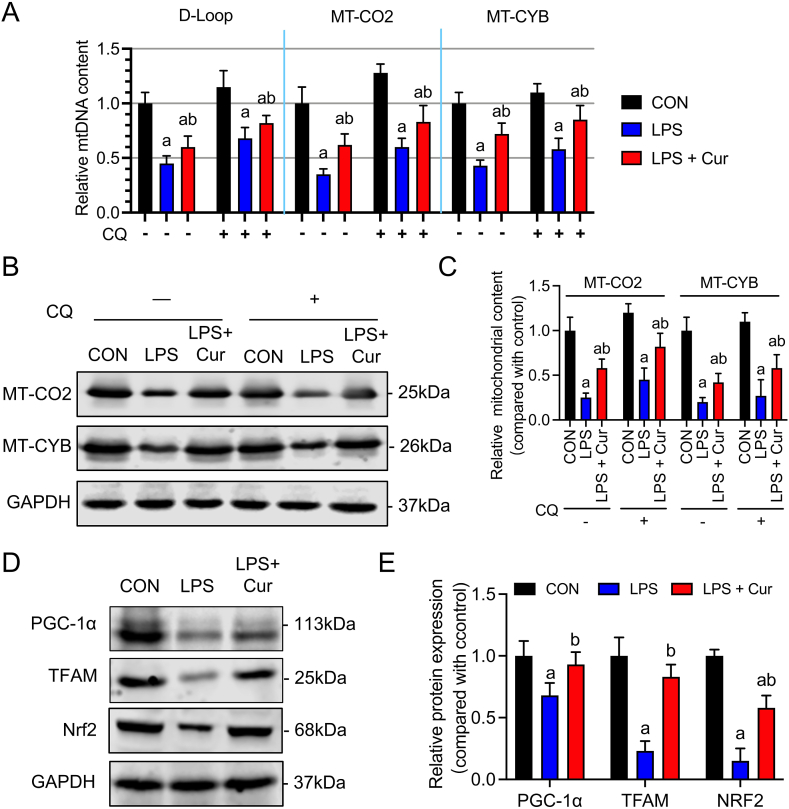
Effects of curcumin on mitochondrial biogenesis in LPS-induced HL-1 cells. **(a)** Quantitative PCR analysis of D-Loop, MT-CO2, and MT-CYB expression. **(b and c)** Immunoblotting analysis of MT-CO2 and MT-CYB expression. **(d and e)** Immunoblotting analysis of PGC-1α, TFAM, and Nrf2 expression. Data are presented as the mean ± standard deviation (n = 3). a, Statistically significant differences (P < 0.05) compared to the control group; b, statistically significant differences (P < 0.05) compared to the LPS group.

PGC-1α and its downstream co-transcription factors Nrf2 and TFAM control the activation of mitochondrial biogenesis [32]. Western blotting showed that LPS significantly inhibited PGC-1α, Nrf2, and TFAM protein expression in HL-1 cells compared to the expression levels in the control group (Fig. 3d and e, Fig, S2), indicating impaired mitochondrial biogenesis. After curcumin treatment, the suppression of PGC-1α, Nrf2, and TFAM was improved (P < 0.05), suggesting that curcumin can improve mitochondrial biogenesis in septic cardiac cells by activating PGC-1α and its downstream co-transcription factors.

### 3.4 Curcumin synergistically regulates DRP1 mitochondrial translocation and the PGC1 pathway after sepsis through SIRT1 activation

SIRT1, a deacetylase, activates PGC-1α by deacetylation, thereby promoting mitochondrial biogenesis [33]. SIRT1 also regulates DRP1 expression and translocation [34]. Western blot analysis revealed that LPS stimulation significantly reduced SIRT1 expression in cardiac cells, whereas curcumin treatment attenuated this effect (P < 0.05) (Fig. 4a, Fig, S3). Curcumin reversed the mitochondrial translocation and reduced DRP1 biogenesis in septic cardiomyocytes.

**Fig. 4:**
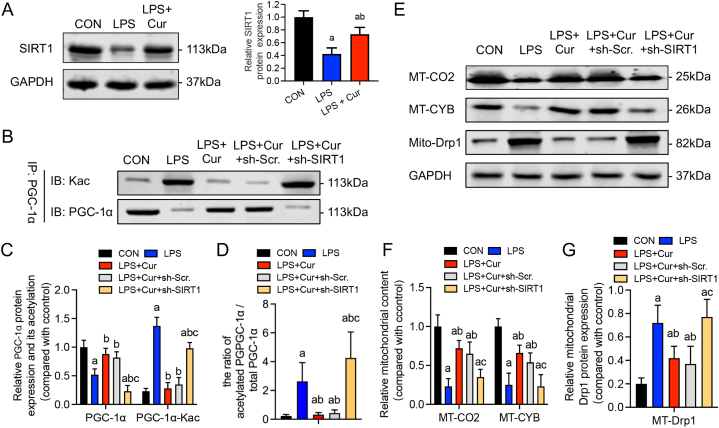
Interfering with SIRT1 expression can reverse the effect of curcumin on mitochondrial biogenesis and DRP1 mitochondrial translocation in LPS-induced HL-1 cells. **(a)** Immunoblotting analysis of SIRT1 in HL-1 cells induced by LPS and treated with curcumin. **(b**–**e)** Immunoblotting analysis of the effects of sh-SIRT1 on PGC-1α **(c)**, MT-CO2 **(D)**, MT-CYB **(d)**, and DRP1 **(e)** expression in HL-1 cells treated with curcumin and LPS for 24 h. Data are expressed as the mean ± standard deviation (n = 3). a, Statistically significant differences (P < 0.05) compared to the control group; b, statistically significant differences (P < 0.05) compared to the LPS group.

We further explored whether SIRT1 regulated these effects by initially silencing SIRT1 in HL-1 cells using shRNA transfection. We then measured the PGC-1α acetylation level using Co-IP. Under LPS stimulation, PGC-1α expression was suppressed, and its acetylation level was enhanced (P < 0.05). Curcumin treatment reversed this effect, increasing PGC-1α expression and inhibiting its acetylation level (P < 0.05). Furthermore, compared to those in the Cur group, additional SIRT1 silencing eliminated the changes in PGC-1α expression and acetylation level induced by curcumin treatment (Fig. 4b and c).

We also assessed the level of mitochondrial biogenesis. Compared to those in the Cur group, SIRT1 silencing significantly decreased the protein expression levels of MT-CO2 and MT-CYB (Fig. 4b and d, Fig, S3). These results suggest that curcumin promotes mitochondrial biogenesis in a SIRT1-dependent manner. Regarding mitochondrial dynamics, further interference with SIRT1 expression significantly suppressed the curcumin-induced reduction in DRP1 mitochondrial translocation, indicating that curcumin inhibits DRP1 mitochondrial translocation in a SIRT1-dependent manner (Fig. 4c and e, Fig, S3).

### 3.5 Curcumin regulates LPS-induced mitochondrial dysfunction in cardiomyocytes via SIRT1-dependent mitochondrial mass regulation

Because mitochondrial quality control directly affects mitochondrial function, we further investigated whether curcumin protects against SCM by regulating mitochondrial function based on changes in the ΔΨm and ROS levels in HL-1 cells among treatment groups. After 24 h of LPS stimulation, the ΔΨm level significantly decreased and ROS production significantly increased compared to those in the CON group. These effects were significantly reversed by curcumin treatment. However, SIRT1 silencing weakened the effects of curcumin on ΔΨm and ROS (P > 0.05) (Fig. 5). These findings suggest that curcumin enhances mitochondrial function and reduces oxidative stress in septic cardiomyocytes by activating SIRT1.

**Fig. 5:**
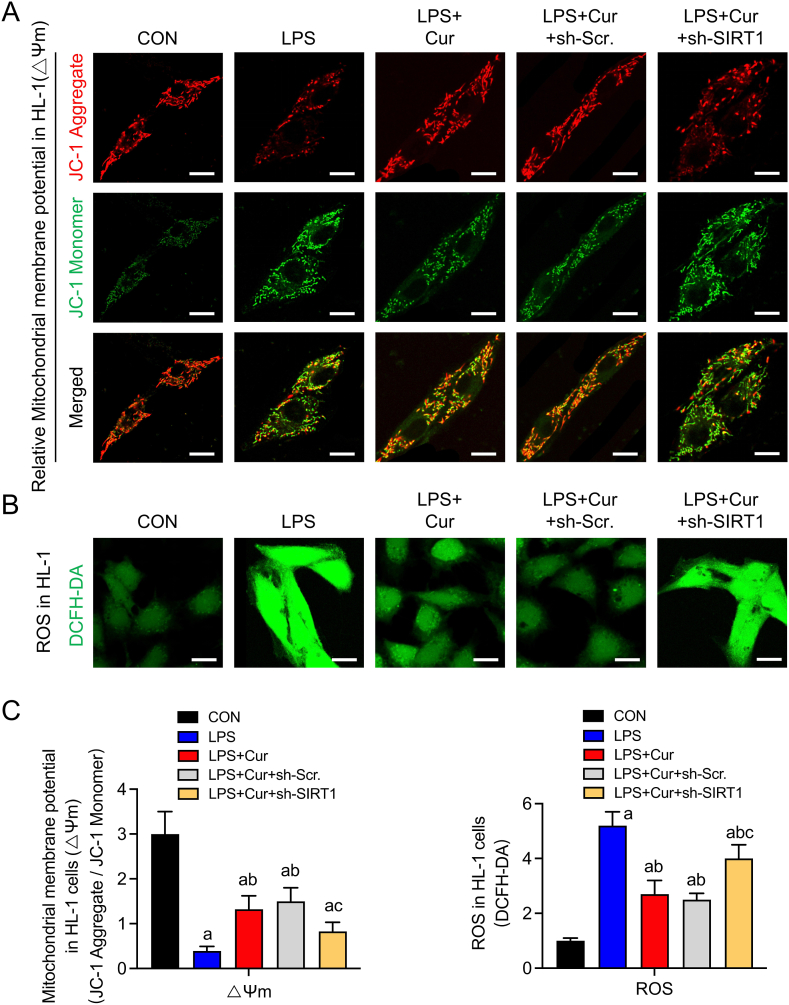
Effect of sh-SIRT1 on curcumin-induced changes in mitochondrial bioenergetics in LPS-induced HL-1 cells. **(a)** Confocal images (63 × magnification; scale bar, 25 μm) showing JC-1 monomers (green fluorescence probe) and JC-1 aggregates (red fluorescence probe) labeling mitochondrial membrane potential (Δψm) in HL-1 cells of each group. **(b)** Confocal images (scale bar, 50 μm) of ROS fluorescence intensity in HL-1 cells of each group. **(c)** Quantification of Δψm and ROS fluorescence intensity. Data are expressed as the mean ± standard deviation (n = 3). a, Statistically significant differences (P < 0.05) compared to the control group; b, statistically significant differences (P < 0.05) compared to the LPS group. (For interpretation of the references to colour in this figure legend, the reader is referred to the Web version of this article.)

## 4 Discussion

The pathology of SCM is primarily caused by mitochondrial dysfunction [35,36]. Using mouse and cell models of LPS-induced sepsis, we discovered that curcumin regulated mitochondrial function by enhancing mitochondrial quality, reversed septic myocardial structural and functional damage, and significantly reduced the levels of the pro-inflammatory cytokines TNF-α, IL-1β, and IL-6. Mechanistically, curcumin induces SIRT1 activation, which deacetylates and activates PGC-1α to increase its expression and promote mitochondrial biogenesis. In contrast, curcumin reduced the mitochondrial translocation of DRP1, decreased excessive mitochondrial fission, and ultimately restored myocardial mitochondrial quality, improving the septic cardiac function and prognosis (Fig. 6). These findings provide crucial evidence supporting the protective effects of curcumin against SCM, with potential therapeutic applications.

**Fig. 6:**
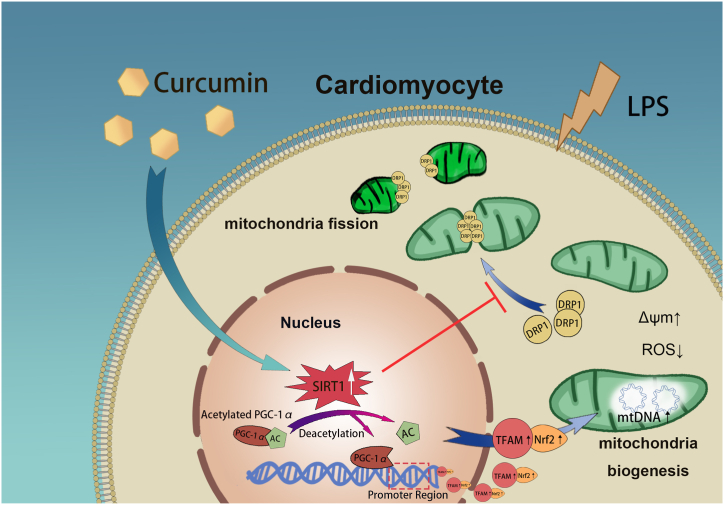
Schematic diagram illustrating the protective effect of curcumin on sepsis-induced cardiac dysfunction via the SIRT1-DRP1/PGC-1α pathway. Sepsis reduces SIRT1 expression in cardiac myocytes, whereas curcumin activates the nuclear deacetylase SIRT1. SIRT1 exerts its protective effects through two pathways [1]: SIRT1 deacetylates and activates PGC-1α, which induces the transcriptional regulation of Nrf2 and TFAM, thereby promoting mitochondrial biogenesis [2]; SIRT1 reduces the translocation of DRP1 from the cytoplasm to mitochondria, thereby reducing mitochondrial fragmentation and restoring mitochondrial morphology. Combining these two pathways protects mitochondrial function, reduces ROS production, increases mitochondrial membrane potential, and ultimately preserves cardiac function.

Curcumin is a traditional Chinese medicine with hypolipidemic, anti-tumor, anti-inflammatory, choleretic, and antioxidant effects and has been widely used to treat cardiovascular diseases [37,38]. Prior studies have explored the cellular protective mechanisms of curcumin, including its ability to reduce the release of pro-inflammatory mediators, such as IL-6, IL-1, and TNF-α, and inhibit the anti-inflammatory effect of NF-κB [23,39,40]. Curcumin has high antioxidant activity, which can protect cell membranes by inhibiting lipid peroxidation [23]. Similar to findings from previous research [3,22], in this study, curcumin demonstrated potent anti-inflammatory antioxidant properties and maintained myocardial function in LPS-induced mouse models. Furthermore, maintaining a healthy mitochondrial network is vital for preserving heart function. Curcumin treatment protects mitochondria in various diseases, including myocardial ischemia-reperfusion injury and heart failure [41,42]. However, previous studies have not confirmed if curcumin alleviates SCM by regulating mitochondrial function. Our study thus provides novel evidence supporting the use of curcumin in the prevention and treatment of SCM.

Sepsis frequently affects the heart, and sepsis-induced cardiac dysfunction is a severe complication characterized by reduced myocardial contractility and cardiac output. Severe cardiac dysfunction may require mechanical ventilation, renal replacement therapy, extracorporeal membrane oxygenation, and other therapies, and it may induce life-threatening conditions, such as decreased blood pressure, inadequate organ perfusion, and pulmonary edema [43]. Therefore, investigating the pathogenesis of SCM and identifying new therapeutic targets are crucial for improving patient prognosis. Accordingly, this study explored the relationship between sepsis-induced cardiac and mitochondrial dysfunction.

Sepsis decreased the mitochondrial biogenesis-related gene expression and increased DRP1-mediated mitochondrial fission, resulting in mitochondrial dysfunction. Our findings are consistent with those of previous studies, including a study showing that sepsis and shock-induced severe ischemia-reperfusion injury activate DRP1-mediated mitochondrial fission, causing myofibrillar protein depletion and cytoskeleton disintegration in myocardial cells [44]. Mitochondrial fragmentation can lead to oxidative stress, and ROS-induced lipid peroxidation can damage mitochondrial membrane integrity, causing the release of pro-apoptotic factors and worsening mitochondrial dysfunction [29]. Additionally, sepsis can reduce mitochondrial biogenesis by causing excessive inflammatory responses and oxidative damage to mtDNA, resulting in energy metabolism disorders and cellular dysfunction [17]. Our study revealed that curcumin treatment enhanced the sepsis-induced excessive mitochondrial fission and inhibited mitochondrial biogenesis. By restoring mitochondrial quality control, curcumin can mitigate the excessive inflammation and myocardial damage caused by sepsis, thereby achieving therapeutic goals.

We focused on the SIRT1-DRP1/PGC-1α signaling pathway to investigate the potential mechanisms underlying curcumin treatment, as this is a pivotal pathway in mitochondrial dynamics and biogenesis. SIRT1 can regulate various protein activities through deacetylation and is involved in mitochondrial quality control processes, such as mitochondrial ROS production [45], mitophagy [46], mitochondrial dynamics [34], and biogenesis [16]. PGC-1α is a crucial transcription factor involved in mitochondrial biogenesis, and its upregulation promotes the transcription of genes involved in this process, improving mitochondrial quality and function [44]. Our findings demonstrate that curcumin upregulates SIRT1 expression, and SIRT1 can activate nuclear PGC-1α activity and expression through deacetylation, promoting mitochondrial biogenesis.

Furthermore, in LPS-stimulated HL-1 cells, curcumin treatment improved the mitochondrial translocation of DRP1, which depended on SIRT1 activation. Although there was no significant change in the DRP1 expression level, its translocation from the cytoplasm to the mitochondria induced mitochondrial division, resulting in fragmented and abnormal mitochondria. These results are supported by other studies showing that SIRT1 could activate the AKT pathway in patients with diabetic myocardial infarction, leading to AKT phosphorylation at the Ser637 site and reducing DRP1 activation and mitochondrial DRP1 expression [47]. Melatonin can protect the hearts of diabetic mice from DRP1-mediated mitochondrial fission in a SIRT1/PGC-1α-dependent manner, and PGC-1α directly regulates *DRP1* expression by binding to its promoter [34]. However, the mitochondrial translocation of DRP1 may be influenced by multiple factors, including DRP1-Ser616 phosphorylation and DRP1-Ser637 dephosphorylation [48,49], DRP1-Thr585/Thr595 glycosylation modification [50], and choline metabolism-mediated mitochondrial membrane phospholipid remodeling [51]. Therefore, the effect of SIRT1 on DRP1 translocation may be related to these mechanisms; however, further validation is required.

Our experimental findings demonstrate that curcumin improves sepsis-induced cardiac dysfunction by reducing mitochondrial fission and promoting mitochondrial biogenesis. However, this study has some limitations. We only investigated the effects of curcumin in animal models and *in vitro* cardiomyocyte cultures. Additionally, the SIRT1-mediated mechanism of regulating the mitochondrial translocation of DRP1 is still unclear.

## 5 Conclusion

In this study, we provide the first demonstration that curcumin significantly improves mitochondrial quality, myocardial dysfunction, and structural damage to the heart and reduces oxidative stress by activating SIRT1 and PGC-1α to promote mitochondrial biogenesis. Moreover, curcumin inhibits the mitochondrial translocation of DRP1 by inhibiting mitochondrial fragmentation in both *in vivo* and *in vitro* models of LPS-induced sepsis. In conclusion, our results suggest that curcumin has potential therapeutic effects on SCM and that the SIRT1-DRP1/PGC-1α-mediated mitochondrial mass regulation pathway may be a novel target for future organ-protective drug development in critical illness. Further studies are needed to confirm the therapeutic effects of curcumin in clinical settings and to determine how SIRT1 regulates the mitochondrial translocation of DRP1.

## Data availability statement

The datasets of this study can be found at https://www.jianguoyun.com/p/DUbfmMoQtMvLChjg3LcFIAA.

## Ethics statement

The experimental procedures undertaken in this study were approved by the Ethics Committee of the Second Affiliated Hospital of Chongqing Medical University. These procedures were performed in accordance with the National Institutes of Health Guide for the Care and Use of Laboratory Animals. Ethical approval number: (2022)222.

## Funding

This work was supported by the 10.13039/501100001809National Natural Science Foundation of China [grant no. 82272252], Chongqing Talents Program [grant no. CQYC202103061], and Kuanren Talents Program of the Second Affiliated Hospital of 10.13039/501100004374Chongqing Medical University. The funders played no roles in the study design; in the collection, analysis, and interpretation of data; in the writing of the report; and in the decision to submit the article for publication.

## CRediT authorship contribution statement

**Dongyao Hou:** Software, Methodology, Formal analysis, Data curation. **Haitang Liao:** Software, Data curation. **Shuai Hao:** Writing – review & editing, Investigation. **Ruixue Liu:** Writing – review & editing, Writing – original draft, Methodology. **He Huang:** Supervision, Conceptualization. **Chenyang Duan:** Funding acquisition, Conceptualization.

## Declaration of competing interest

The authors declare the following financial interests/personal relationships which may be considered as potential competing interests: Chenyang Duan reports financial support was provided by 10.13039/501100001809National Natural Science Foundation of China. Chenyang Duan reports financial support was provided by Chongqing Talents Program. Chenyang Duan reports was provided by The Second Affiliated Hospital of Chongqing Medical University. If there are other authors, they declare that they have no known competing financial interests or personal relationships that could have appeared to influence the work reported in this paper.

## Figures and Tables

**figs1:**
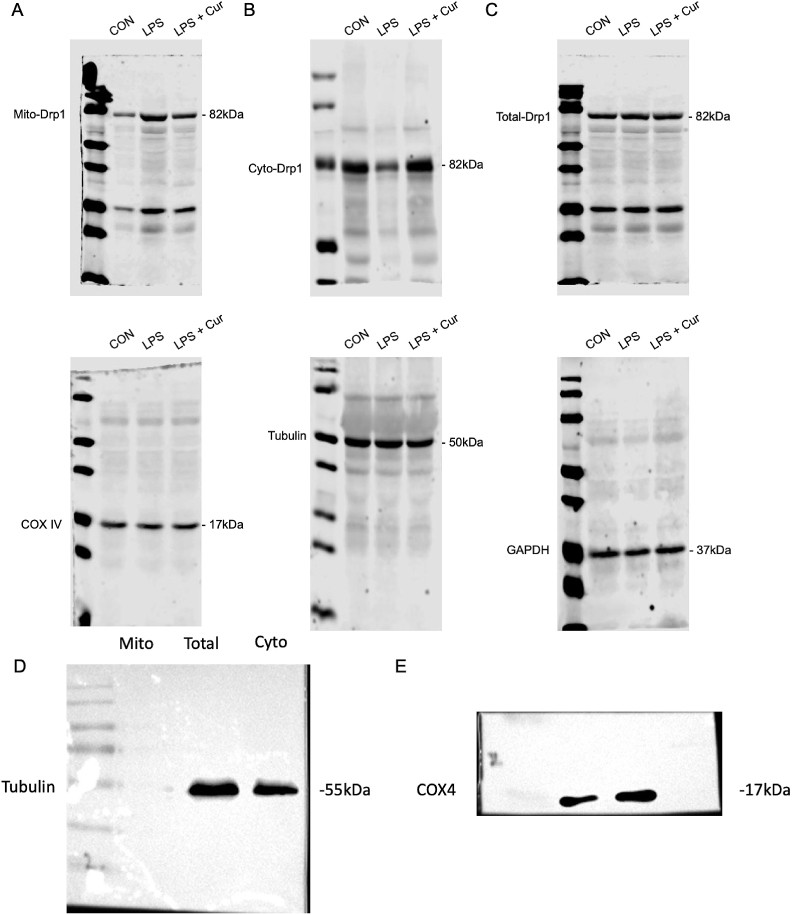


**figs2:**
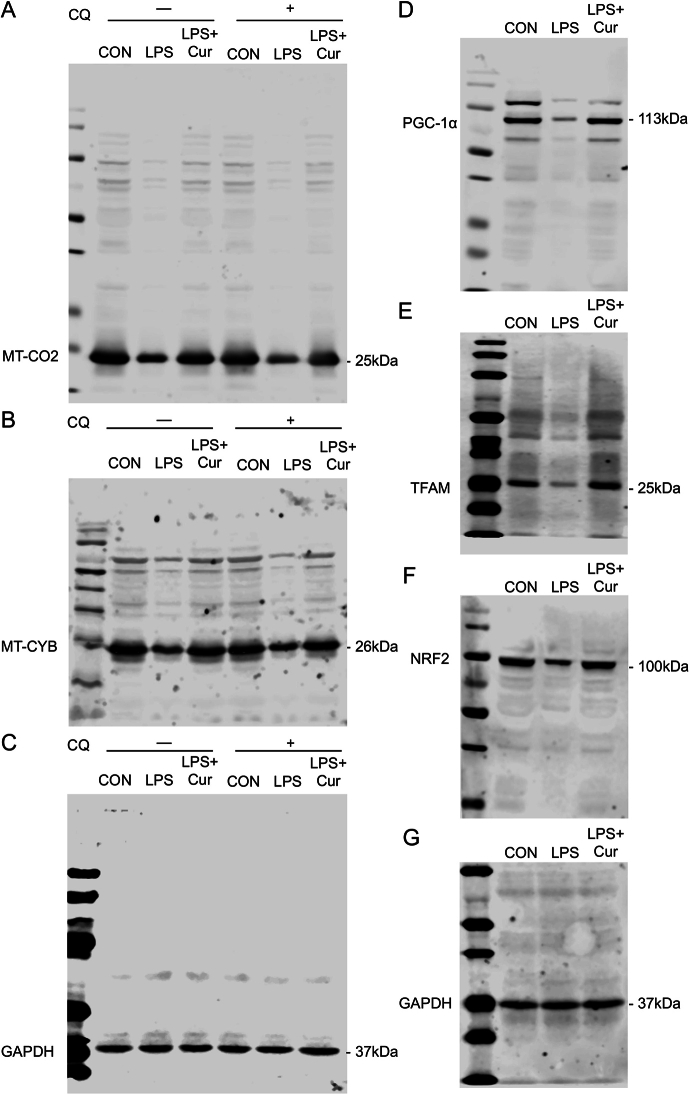


**figs3:**
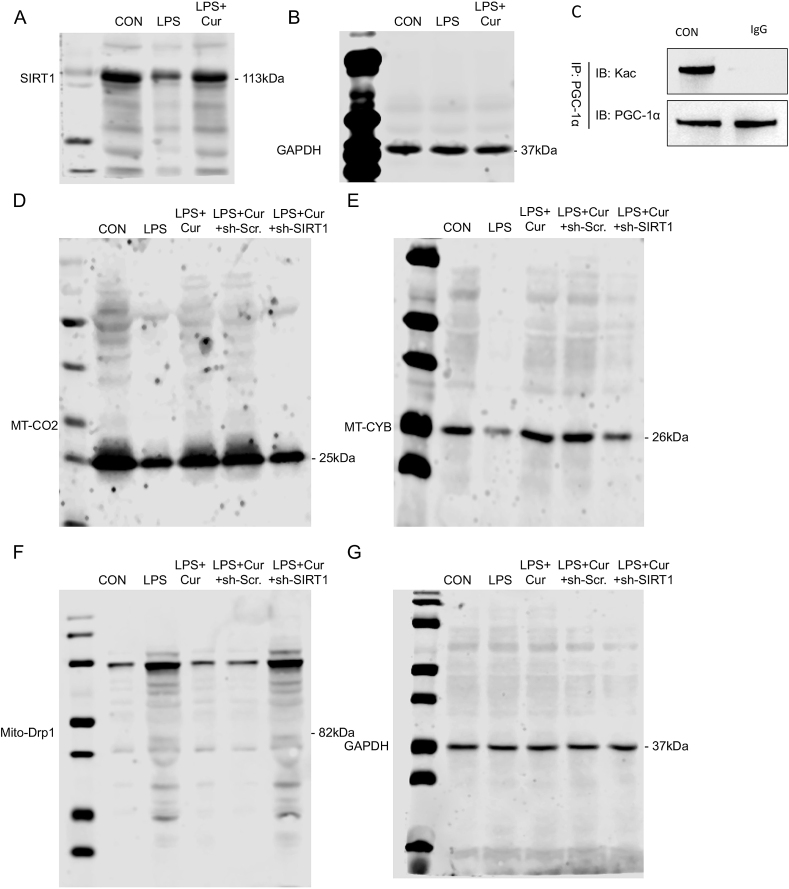


**figs4:**
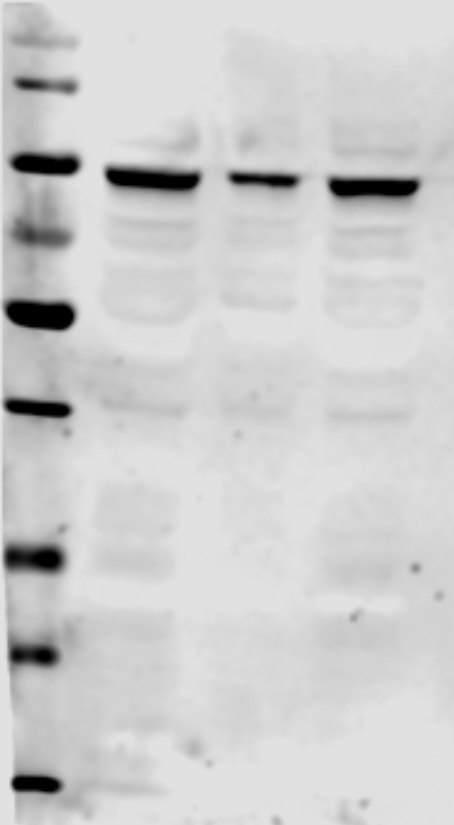


**figs5:**
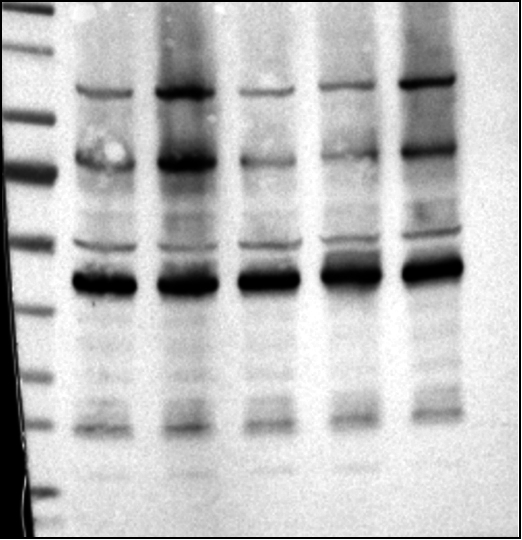


**figs6:**
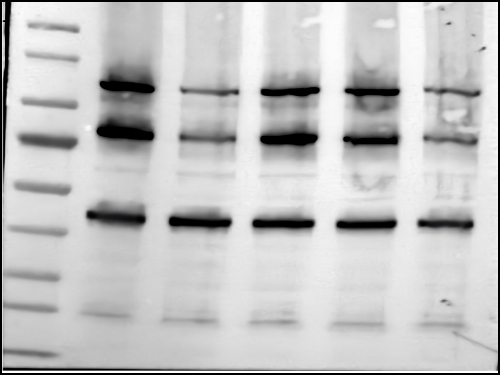


**figs7:**
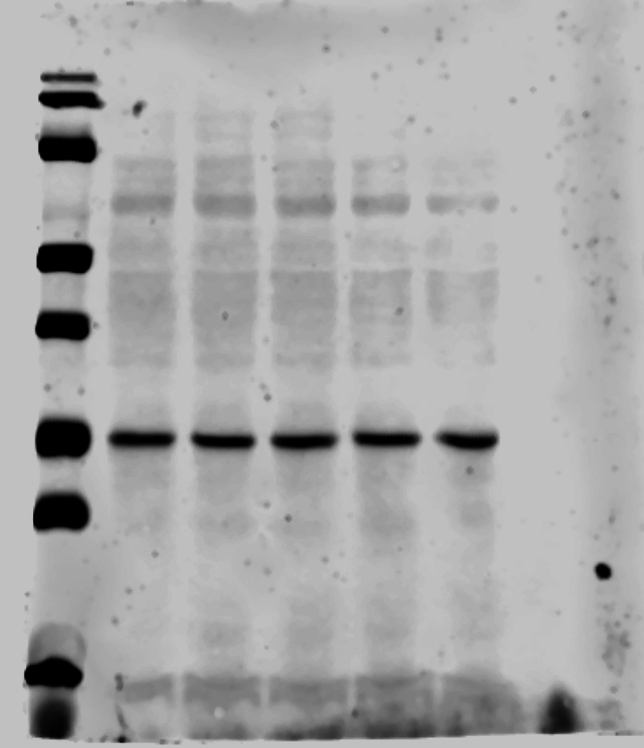


**figs8:**
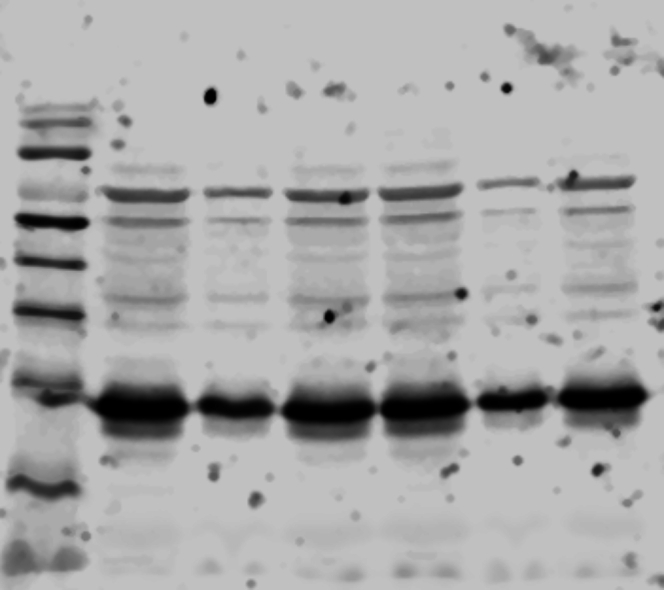


**figs9:**
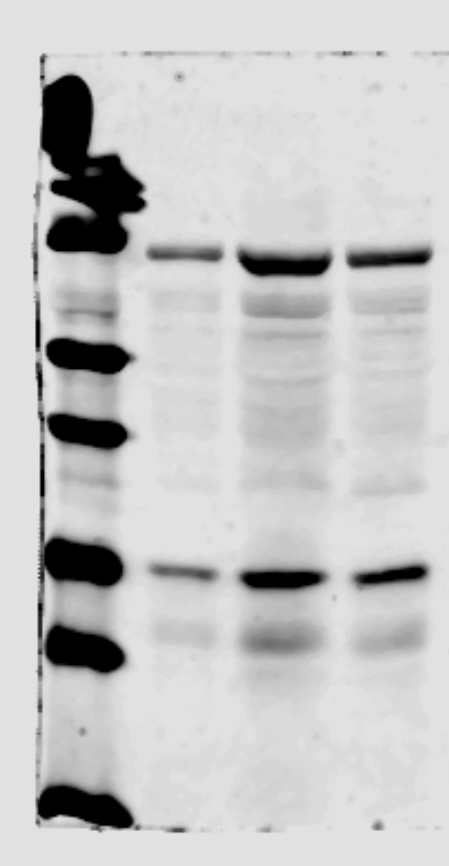


**figs10:**
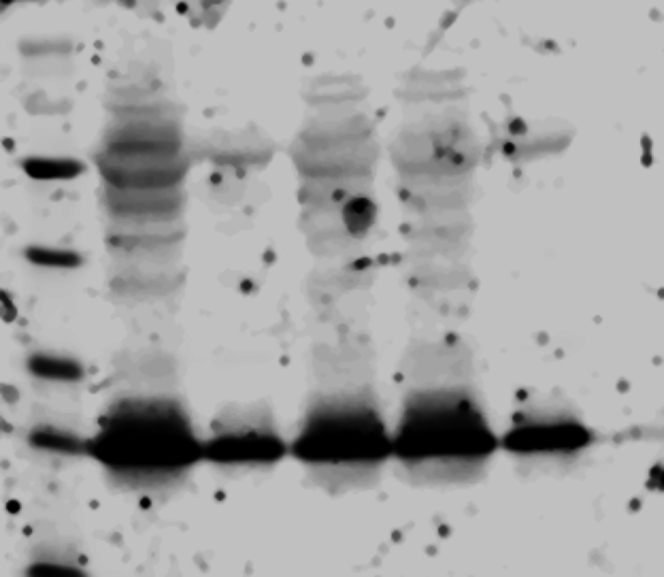


**figs11:**
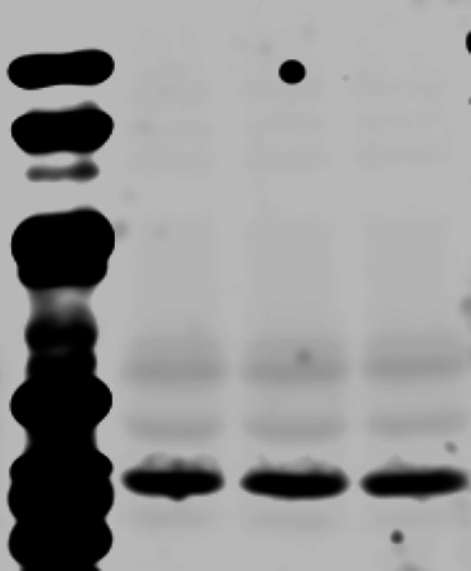


**figs12:**
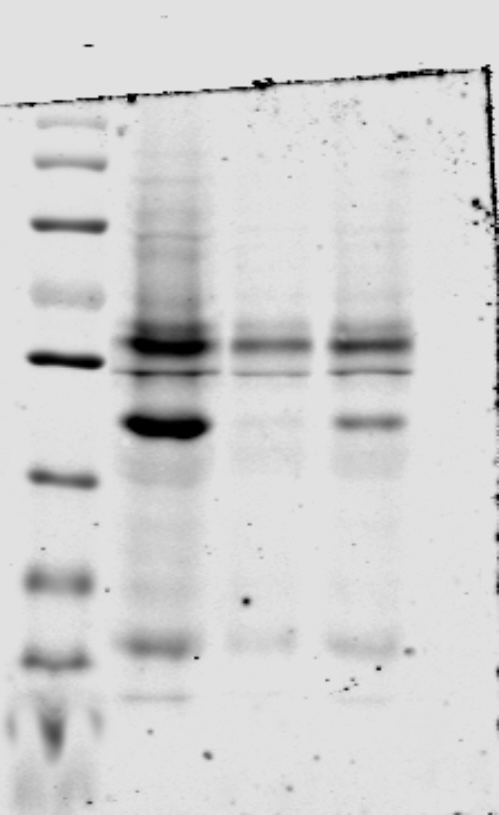


**figs13:**
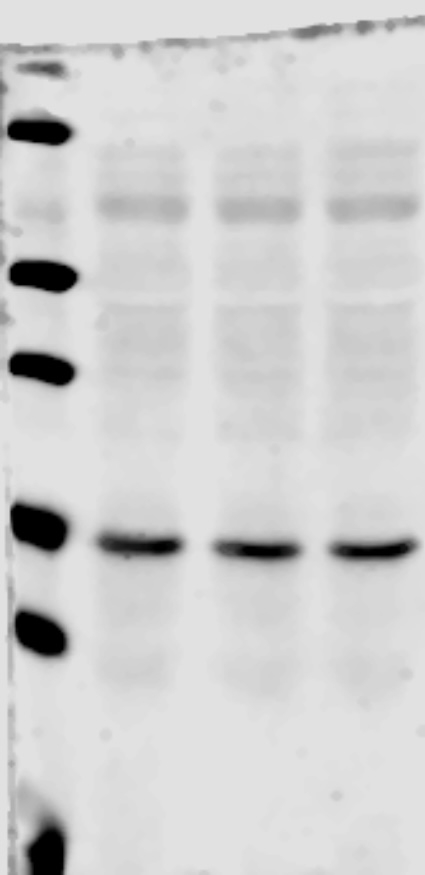


**figs14:**
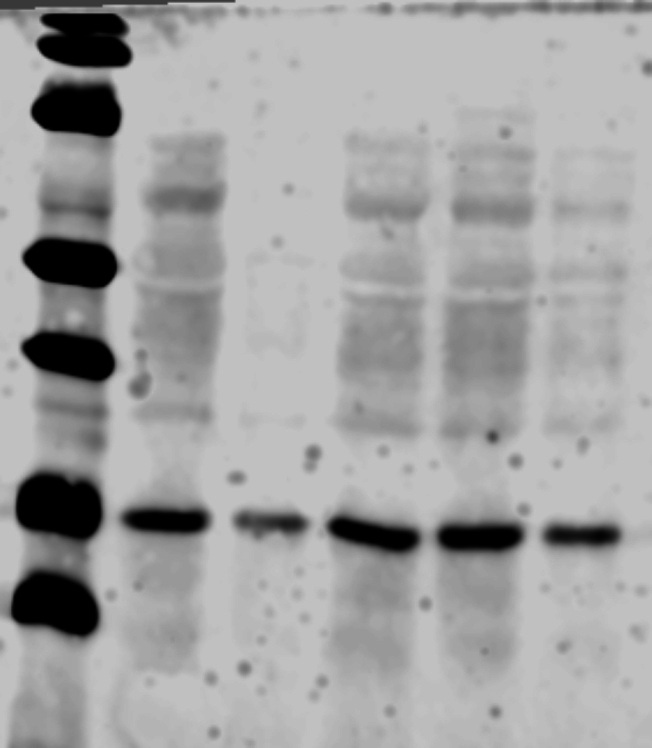


**figs15:**
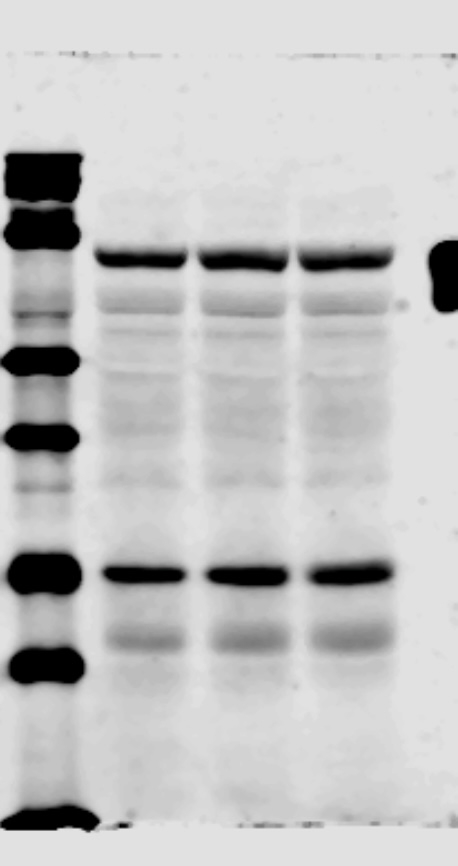


**figs16:**
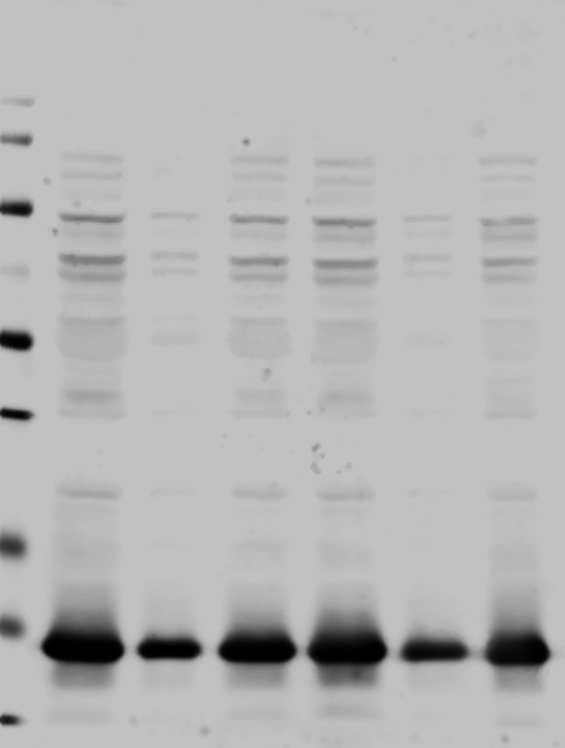


**figs17:**
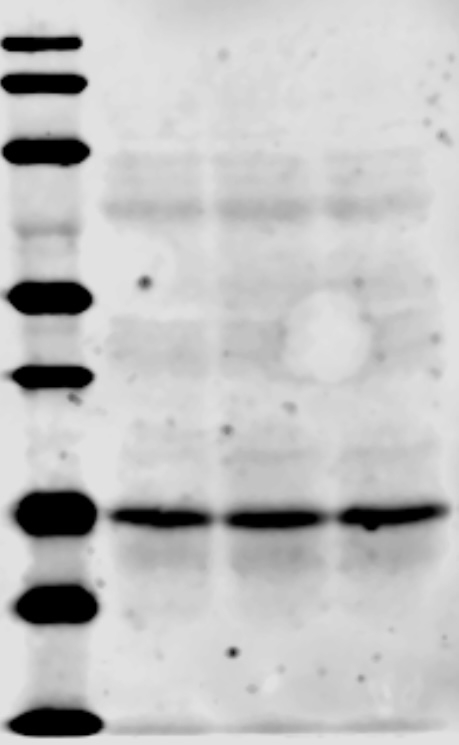


**figs18:**
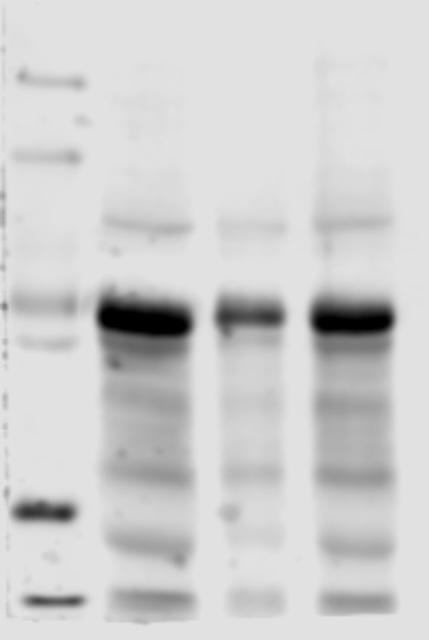


**figs19:**
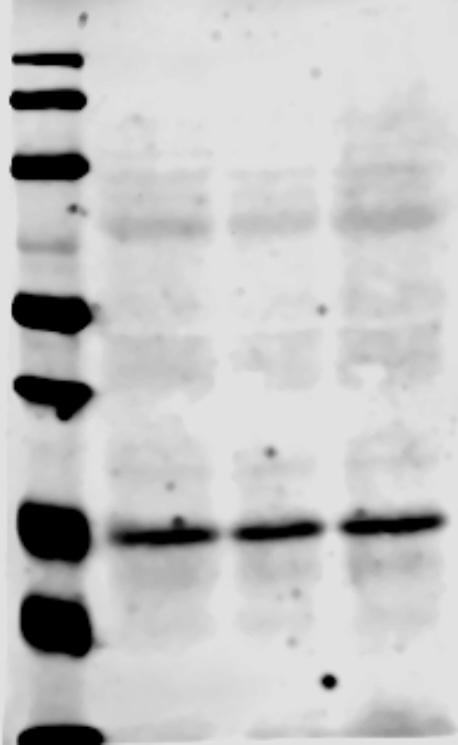


**figs20:**
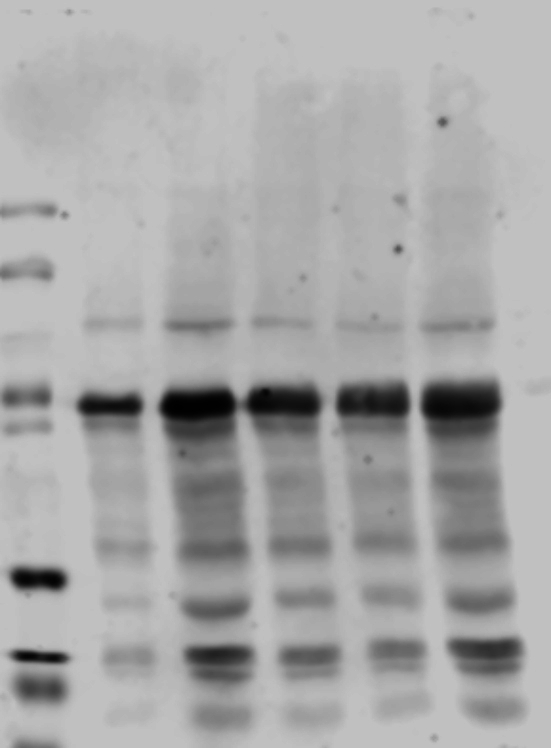


**figs21:**
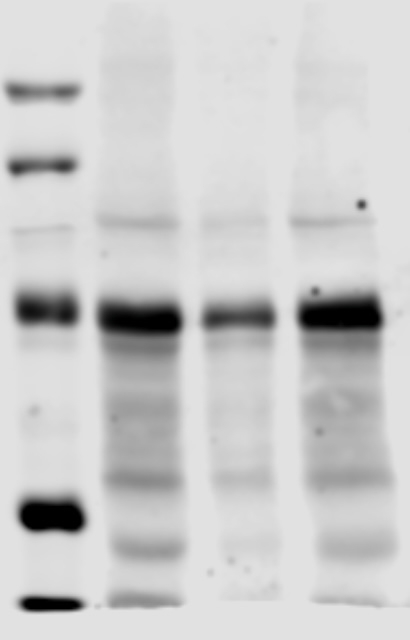


**figs22:**
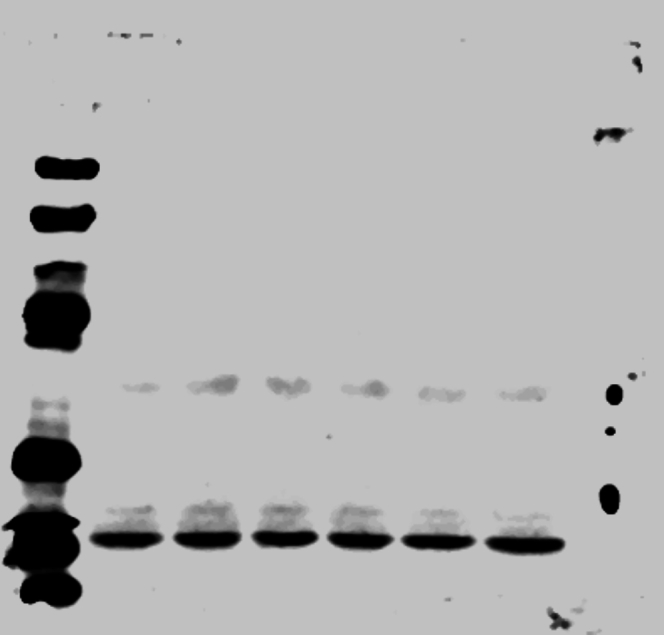


**figs23:**
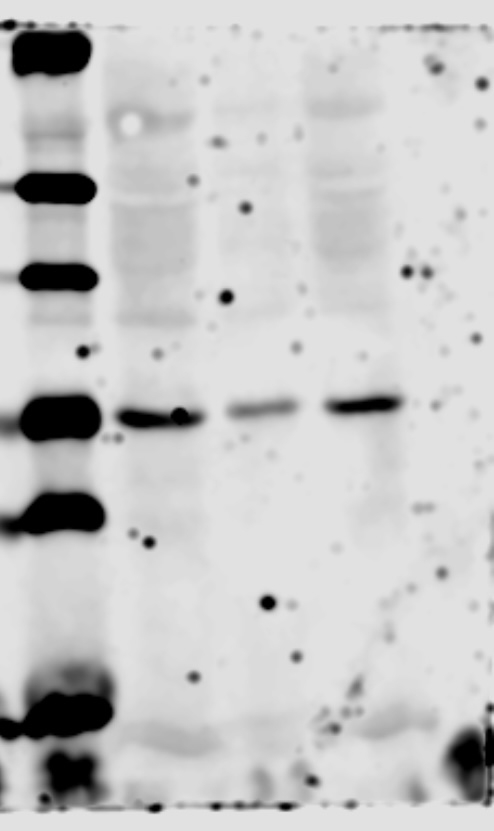


**figs24:**